# The laminA/NF-Y protein complex reveals an unknown transcriptional mechanism on cell proliferation

**DOI:** 10.18632/oncotarget.12914

**Published:** 2016-10-26

**Authors:** Lucia Cicchillitti, Isabella Manni, Carmine Mancone, Giulia Regazzo, Manuela Spagnuolo, Tonino Alonzi, Fabrizio Carlomosti, Maria Dell’Anna Lucia, Giulia 'Omo Dell, Mauro Picardo, Paolo Ciana, Maurizio C Capogrossi, Marco Tripodi, Alessandra Magenta, Maria Rizzo Giulia, Aymone Gurtner, Giulia Piaggio

**Affiliations:** ^1^ Department of Research, Advanced Diagnostics and Technological Innovation, SAFU Unit, Translational Research Area, Regina Elena National Cancer Institute, 00144 Rome, Italy; ^2^ National Institute for Infectious Diseases L. Spallanzani, IRCCS, Department of Epidemiology and Preclinical Research, 00149 Rome, Italy; ^3^ Department of Cellular Biotechnologies and Haematology, Istituto Pasteur Italia, Fondazione Cenci Bolognetti, Sapienza University of Rome, 00161 Rome, Italy; ^4^ Department of Research, Advanced Diagnostics and Technological Innovation, Genomic and Epigenetic Unit, Translational Research Area, Regina Elena National Cancer Institute, Rome, Italy; ^5^ Fondazione Luigi Maria Monti, Istituto Dermopatico dell’Immacolata-IRCCS, Laboratorio di Patologia Vascolare, 00167 Rome, Italy; ^6^ Cutaneous Physiopathology and Integrated Center of Metabolomics Research, San Gallicano Dermatologic Institute, IRCCS, 00144 Rome, Italy; ^7^ Department of Oncology and Hemato-Oncology and Department of Pharmacological and Biomolecular Sciences, University of Milan, 20133 Milan, Italy; ^8^ Center of Excellence on Neurodegenerative Diseases, Department of Oncology and Hemato-Oncology, University of Milan, 20133 Milan, Italy

**Keywords:** transcription, proliferation, cell cycle, euchromatin, nuclear lamina

## Abstract

Lamin A is a component of the nuclear matrix that also controls proliferation by largely unknown mechanisms. NF-Y is a ubiquitous protein involved in cell proliferation composed of three subunits (-YA -YB -YC) all required for the DNA binding and transactivation activity. To get clues on new NF-Y partner(s) we performed a mass spectrometry screening of proteins that co-precipitate with the regulatory subunit of the complex, NF-YA. By this screening we identified lamin A as a novel putative NF-Y interactor. Co-immunoprecipitation experiments and confocal analysis confirmed the interaction between the two endogenous proteins. Interestingly, this association occurs on euchromatin regions, too. ChIP experiments demonstrate lamin A enrichment in several promoter regions of cell cycle related genes in a NF-Y dependent manner. Gain and loss of function experiments reveal that lamin A counteracts NF-Y transcriptional activity. Taking advantage of a recently generated transgenic reporter mouse, called MITO-Luc, in which an NF-Y–dependent promoter controls luciferase expression, we demonstrate that lamin A counteracts NF-Y transcriptional activity not only in culture cells but also in living animals. Altogether, our data demonstrate the occurrence of lamin A/NF-Y interaction and suggest a possible role of this protein complex in regulation of NF-Y function in cell proliferation.

## INTRODUCTION

NF-Y is an heterotrimeric transcriptional factor composed of 3 proteins, the regulatory subunit NF-YA, NF-YB and NF-YC. The three proteins are transcribed from independent genes highly conserved from yeast to human. All 3 proteins are necessary for NF-Y binding to the target DNA sequence, the CCAAT-box. The NF-YA subunit contain a DNA binding domain, an activation domain and the binding site for the dimer NF-YB/-YC. The NF-YB and -YC subunits contain histone-like domains [1]. A bioinformatic analysis of promoters of cell-cycle regulatory genes demonstrated that CCAAT box is one of the most represented cis-regulatory elements in particular on promoters regulated during the G2/M transition [2]. In agreement, growing number of experiments demonstrate that the NF-Y complex is a key regulator of cell-cycle genes responsible for cell progression in all cell cycle phases among which are mitotic cyclin complexes [3–10]. Taken together, these studies demonstrate that NF-Y is a major player in the regulation of proliferation.

It has been reported the presence of two major NF-YA isoforms, “long” and “short”, the short isoform lacking a 28-amino acid within the NF-YA amino-terminal domain [11]. Expression of NF-YA subunit is tightly regulated in normal cells during the cell cycle [4], moreover, in agreement with the key role of NF-Y on proliferation, the abrogation of expression of its regulatory subunit NF-YA plays an important role in downregulating cell-cycle genes in differentiated cells [5, 7–10]. Previous studies have used a loss of function approach in order to study the biological role of NF-Y. The overexpression of a NF-Y dominant negative protein in mouse fibroblasts leads to a retardation of cell growth [12]. The knock out of the NF-YA subunit in mice leads to embryo lethality; moreover, knock out of the NF-YA gene in mouse embryonic fibroblasts leads to a cell cycle arrest with a consequent inhibition of cell proliferation [13–14]. Abundant evidences indicate that NF-Y plays a role in carcinogenesis. We have demonstrated that NF-Y regulates transcription of several genes upon DNA damage [15, 16], and NF-Y overexpression increased cell proliferation in cell expressing endogenous mutant p53. Next, we have described an *in vivo* NF-Y/mutant p53 complex able to increases DNA synthesis, in a NF-YA dependent manner [8, 17]. Clinical studies have revealed that increased expression of NF-Y target genes correlates with poor prognosis in multiple cancers [8, 18]. Analysis of transcriptome profiles across human cancers revealed the involvement of NF-Y in cancer-associated pathways [19]. In agreement with its wide involvement on human cancers, we have described that NF-Y interacts with different partners. Indeed, we have shown that in normal cells NF-YA binds to deacetylase enzymes (HDACs) while in transformed cells the acetylase p300 is preferentially recruited [8–9]. Although some NF-Y interactors are already known, several partners through which NF-Y exerts its role still need to be characterized.

The major components of the nuclear lamina are lamins. These type V intermediate filament (IF) proteins play important roles in nuclear architecture, mechanosignaling [20] and chromatin dynamics [21], and impact on stem cell proliferation and differentiation [22, 23]. Disruption of one or more of these functions due to lamin mutations cause a group of inherited diseases affecting various tissues and organs or causing accelerated ageing [24]. In mammal exist four lamins isoforms: A-type lamins, counting lamin A and lamin C, and B type lamins, including B1 and B2. Lamin A and lamin C, encoded by *LMNA* gene, are expressed only in differentiated cells, while Lamin B1 and lamin B2, encoded by *LMNAB1* and *LMNAB2* genes, are expressed throughout development. Prelamin A (the precursor of lamin A protein) and lamin C are produced by an alternative splicing within exon 10. The two proteins differ in the carboxyterminal domain where the human lamin A (646aa) contains 80 unique amino acids and lamin C (572aa) contains 6 unique amino acids. It has been shown that lamin A/C stabilizes the nuclear lamina and chromatin, preventing DNA breaks and favouring epigenetic stabilization.

The nuclear lamina interacts with large genomic regions, called lamina-associated domains (LADs). LADs are often located in repressive chromatin structures that appear principally at the nuclear periphery [25, 26]. Besides the well characterized localization at nuclear membranes, lamins display also a nucleoplasmic localization with distinct roles [27–30]. It has been demonstrated that the two isoforms, lamin A and C, participate, at least in part, to distinct networks in the nuclear lamina [31].

Lamins A and C are implicated in epigenetics, heterochromatin organization and are shown to complex with histones and key regulator of transcription such as pRB (retinoblastoma-associated protein), MOK2 (zinc finger transcription repressor), several components of the Pol II (RNA polymerase II) complex [32]. Lund et al have already demonstrated that lamin A and C can associate with euchromatic regions [33, 34]. Lamin A expression is downregulated or absent in cells that are highly proliferative, including various human malignancy [35]. Loss of lamin A expression has been reported for colon cancer, cervical cancer, lung cancer, prostate cancer, gastric cancer, ovarian cancer and leukemia and lymphoma [35–39]. Moreover, the lamin A knock down increase the proliferative potential of cells and impairs cell cycle arrest induced by contact inhibition [40]. Recent data highlight the specific functions of a small pool of lamina-independent A-type lamins, located throughout the nucleoplasm, in the regulation of early tissue progenitor, cell proliferation and commitment [41, 42].

Using a combination of biochemical, cell biology and molecular imaging techniques, we demonstrate here that NF-Y, a master regulator of cell proliferation, forms a complex with a component of the nuclear lamina, lamin A. This interaction impacts on the expression of NF-Y target cell cycle regulatory genes and consequently cell proliferation.

## RESULTS

### NF-Y interacts *in vivo* with lamin A

**Figure 1:**
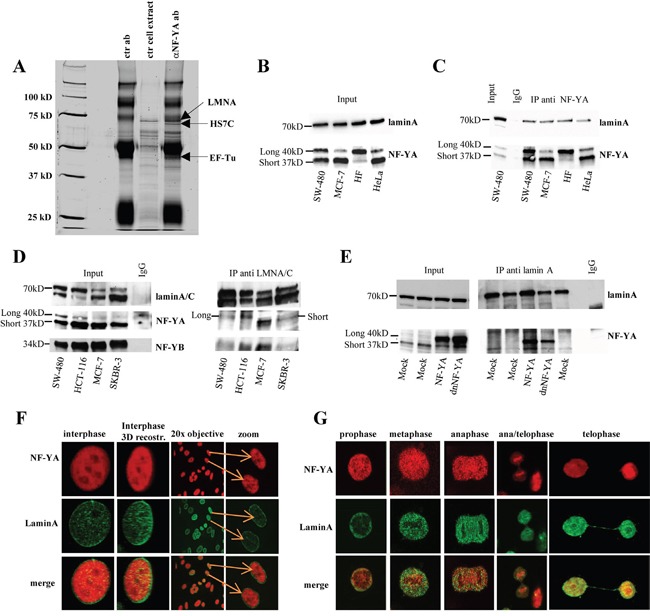
Analysis of the occurrence of lamin A/NF-Y complex in several cell lines **A.** Lamin A interacts with NF-YA. Silver-stained SDS-PAGE gel of the immunoprecipitates with the indicated antibodies from SKBR3 cells. Arrow indicates proteins identified by mass spectrometry specifically associated to NF-YA. ctr cell extract, cellular extracts incubated with protein G-Sepharose without antibody; ctr ab, anti-NF-YA polyclonal antibody-protein-G complexes without cellular extracts **B.** Whole cell lysates from several cell lines were analyzed by western blotting with anti-NF-YA and lamin A antibodies. **C.** Whole cell lysates from several cell lines were immunoprecipitated with an antibody against NF-YA, and western blotting was performed with antibodies to lamin A and NF-YA. **D.** Whole cell lysates from several cell lines were immunoprecipitated with an antibody against lamin A/C, and western blotting was performed with an antibody anti lamin A/C, NF-YA (monoclonal) and NF-YB. As a reference, 1/20 of whole cell extract used in the immunoprecipitations was loaded (input). As negative control, in (C) and (D) were used SW480 cell lysates immunoprecipitated with an anti-IgG antibody. **E.** Immunoprecipitation experiments were performed with whole cell lysates produced from SW480 cells transiently transfected with NF-YA (NF-YA) or a dominant negative mutant of NF-YA subunit (dnNF-YA), or with the empty vector (mock), using antibodies against LaminA. In panel B, C, D, E Long 40 kD means long isoform of NF-YA subunit and Short 37 kD short isoform of NF-YA subunit. **F.** Confocal analysis performed on proliferating SW480 cells using antibodies against NF-YA (tritc) and lamin A (fitc). Different optical fields are shown. Colocalization (yellow) of endogenous NF-YA (red) and lamin A (green) was analyzed by indirect immunofluorescence combined with Confocal Scanning Laser Microscopy. Confocal analysis of single optical section is shown. Different optical fields are shown. **G.** Confocal analysis on mitotic SW480 cells using antibodies against NF-YA (tritc) and lamin A (fitc). Colocalization (yellow) of endogenous NF-YA (red) and lamin A (green) was analyzed by indirect immunofluorescence combined with Confocal Scanning Laser Microscopy. The images have been collected with a 60x oil objective. (Long 40kD) long isoform of NF-YA subunit; (Short 370kD) short isoform of NF-YA subunit.

To get clues on NF-Y function(s) in cancer cells, we performed a mass spectrometry screening of a pool of proteins that co-precipitate with the long NF-YA isoform overexpressed in human breast cancer, SKBR3 cells. By this screening we identified lamin A, but not lamin C, as a novel putative NF-YA interactor (Figure 1A, supplementary Table S1).

The occurrence of laminA/NF-Y interaction was also validated by coimmunoprecipitation experiments between endogenous proteins. As already described it has been reported the presence of two NF-YA isoforms, “long” and “short”. As shown in Figure 1B, both lamin A and the two NF-YA isoforms are expressed in all tested cell lines. Lysates from these growing cells were immunoprecipitated with an antibody against NF-YA and subjected to western blot analysis. Endogenous lamin A, but not lamin C, coimmunoprecipitated with both differentially spliced forms of endogenous NF-YA protein in all cell culture lines (Figure 1C).

Reciprocal immunoprecipitation experiments performed with anti-lamin A/C antibody further validated the occurrence of endogenous lamin A/NF-YA in all tested cell lines (Figure 1D). Apparently, lamin A differently immunoprecipitates the two isoforms depending on cell lines. However, using in WB a different antibody against NF-YA we observed a more similar lamin A immunoprecipitated NF-YA pattern (Supplementary Figure 1A) clearly indicating that the difference observed in Figure 1D could be due to the anti- NF-YA antibody used in WB. As expected NF-YB subunit is present in the complex, too. As shown in Figure 1D and in Supplementary Figure 1B, an antibody against NF-YB protein immunoprecipitated lamin A, further confirming the involvement of this subunit in the complex and thus indicating that lamin A associates with the NF-Y complex and not with the NF-YA subunit alone. Transfection with plasmid encoding the long form of NF-YA in SW-480 cells confirmed this result (Figure 1E). We also overexpressed the dominant negative mutant of NF-YA subunit (dnNF-YA) that carrying a triple aminoacid substitution in the DNA binding domain does not bind DNA and impairs NF-YA transcriptional activity [43]. Results demonstrate that dnNF-YA mutant form is still able to efficiently bind lamin A thus suggesting that the DNA binding domain of NF-YA is not necessary for its interaction with lamin A (Figure 1E).

To demonstrate the specificity of lamin A/NF-YA interaction we evaluated the interaction of lamin A with p53, a well known NF-Y interactor. The results presented in supplementary Figure 1C demonstrate that lamin A does not interact with p53, thus confirming the interaction specificity between lamin A and NF-YA. As expected, NF-YA immunoprecipitates p53 [8]. These results indicate the specificity of the association of lamin A with NF-YA under our experimental conditions and suggest that p53 may be not involved in the formation of the laminA/NF-Y complex. Confocal analysis results obtained on several proliferating cells are compatible with lamin A/NF-YA interaction both in interphase (Figure 1F and Supplementary Figure 1D) and in different mitosis phases (Figure 1G). Interestingly, we observed a colocalization of lamin A and NF-YA both in the nucleoplasm and in the nuclear lamina. Taken together, our data reveals the occurrence of a lamin A/NF-Y nuclear complex.

### NF-YA localizes in lamin enriched nuclear fractions

**Figure 2:**
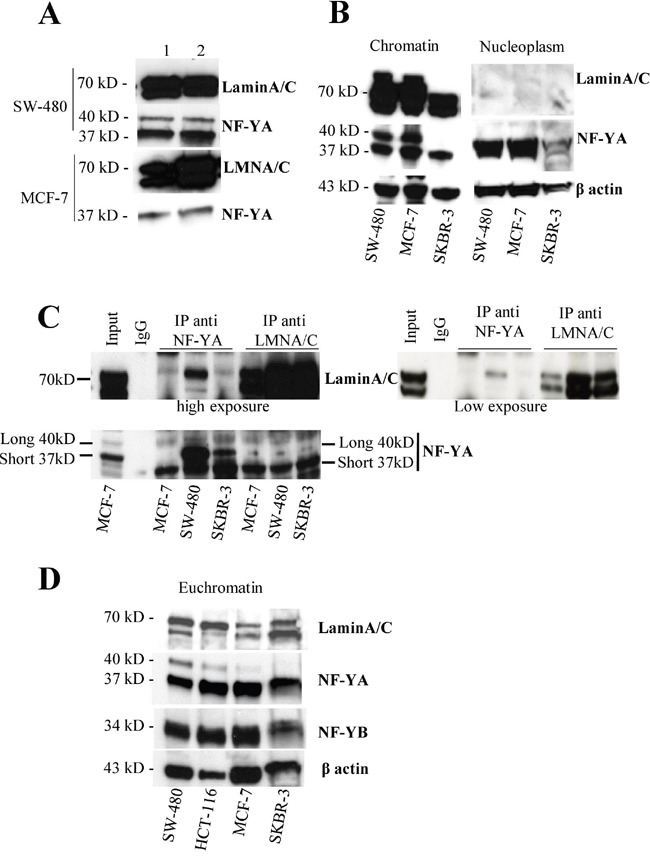
NF-Y localizes in lamin-enriched nuclear fraction and associates with lamin A in the chromatin fraction **A.** Western blotting of soluble (1) and insoluble (2) lamin enriched fractions obtained following the procedure described in Supplementary Figure 2A from SW480 and MCF7 cell lines using anti-NF-YA or -LaminA antibodies. **B.** Chromatin and nucleoplasm fractions obtained following the procedure described in Supplementary Figure 3A from SW480 and MCF7 cell lines were subjected to western blotting analysis using anti-NF-YA or -LaminA antibodies. β actin was used as loading control. **C.** Immunoprecipitation experiments using chromatin fraction isolated from MCF7, SW480 and SKBR3 cell lines obtained following the procedure described in Supplementary Figure 3A using anti-NF-YA and lamin A antibodies followed by western blotting analysis using antibodies against the indicated proteins. **D.** Euchromatin fractions were produced by digestion of chromatin with Micrococcal nuclease as described in Supplementary Figure 3. These fractions were loaded onto a SDS polyacrylamide gel and analyzed by western blotting using the antibodies against the indicated proteins.

To study the function of a complex often is critical the identification of cellular compartment (s) where the interaction between the two proteins occurs. To get clues on lamin A and NF-YA physical interaction, we isolated different lamin enriched nuclear fractions (Supplementary Figure 2A). We isolated nuclei in hypotonic buffer. Firstly, we verified the presence of lamin A by western blot on resuspended nuclei (Supplementary Figure 2B). We treated isolated nuclei with moderate-salt buffer to solubilize most nuclear proteins, and then pelleting to obtain a lamin-enriched pellet. The lamin-enriched pellet was solubilized by high pH and high detergent and, upon centrifugation, we separated soluble lamins from insoluble pellet [44]. The two fractions were studied for the presence of lamin A and NF-YA. As expected, both lamin A and C were present on soluble lamin-enriched fractions and they are still present on insoluble pellet. Interestingly, NF-YA was found both in the lamin-enriched fractions and on insoluble pellet thus suggesting NF-YA as a nucleoskeletal protein (Figure 2A).

Recent data support a role for lamin A in gene regulation through its interaction with chromatin [45]. Thus we asked whether the interaction of lamin A and NF-YA occurs on chromatin. To this purpose, we generated chromatin and nucleoplasm fractions from different cell lines. We isolated nuclei in isotonic-sucrose based buffer and we separated nucleoplasm and chromatin (Supplementary Figure 3A). As shown in Figure 2B, both NF-YA and lamin A were found in the chromatin fraction. NF-YA was present in nucleoplasm deprived of chromatin while lamin A was almost undetectable in this fraction. Next, we performed reciprocal co-immunoprecipitation experiments using chromatin fractions from several cell lines. The antibody against NF-YA immunoprecipitates lamin A and viceversa in all tested cell lines (Figure 2C). Again, with this experiment we confirmed NF-YA binding with lamin A, but not with lamin C. All together these results strongly reinforce the thesis that lamin A/NF-YA association occurs on chromatin, too.

### Lamin A is enriched in promoter regions encompassing CCAAT boxes

To start to investigate whether lamin A localizes on euchromatin, where NF-Y plays a key role as transcription factor, we first isolated euchromatin fraction from several cell lines (Supplementary Figure 3A). To this purpose, we subjected the above isolated chromatin to mild MNase digestion to separate euchromatin, more accessible to MNase digestion. A time course for MNase digestion up to 30 minutes was performed on chromatin from SW-480 and MCF-7 cells in order to determine the minimal incubation time necessary for NF-YA extraction in the euchromatin fraction. After 5 minutes NF-YA and lamin A were both detected in the supernatant (Supplementary Figure 3B).

Based on this result, we digested for 5 minutes chromatin from several cell lines and analyzed it by agarose gel (Supplementary Figure 3C). In all employed cell lines we observed an enrichment of mono-, di- and tri-nucleosomes thus indicating an enrichment of euchromatin in these fractions. Interestingly, by western blot we demonstrated that NF-YA and lamin A are present in this fraction in all tested cell lines (Figure 2D and Supplementary Figure 3D). Taken together the results obtained so far demonstrate the occurrence of a lamin A/NF-Y interaction on chromatin and the two proteins localize on the same open chromatin fraction thus suggesting the possibility that they cooperate on gene regulation.

**Figure 3:**
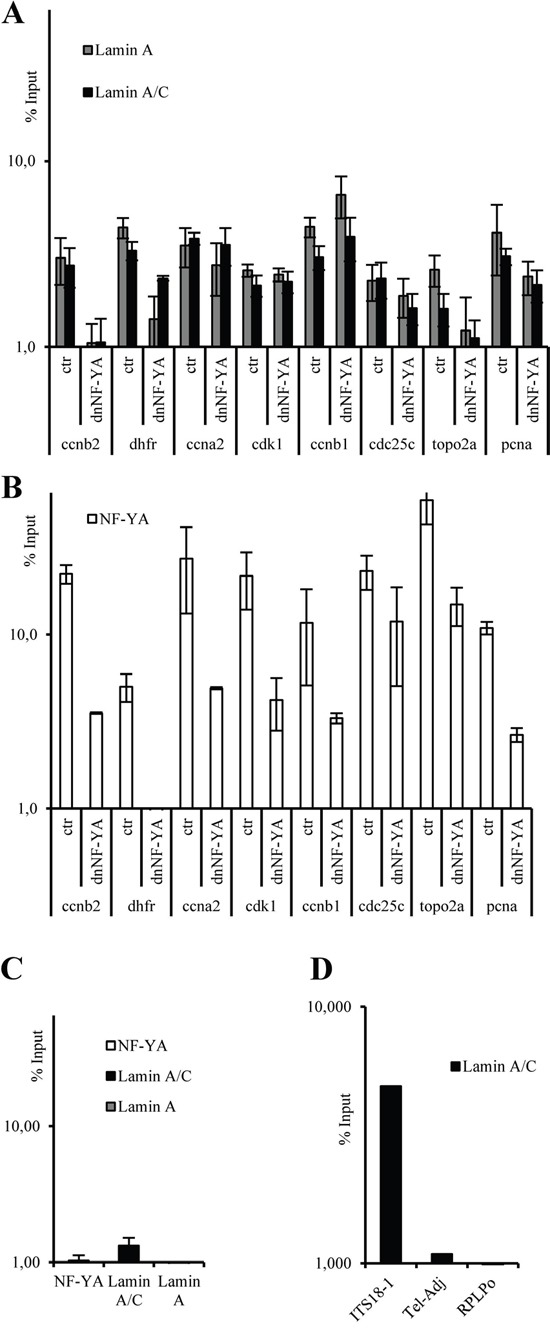
ChIP-qPCR analysis on CCNB2, DHFR, CCNA2, CDK1, CDC25C, TOPO2A, and PCNA promoters with anti-lamin A/C and lamin A antibodies A. and anti-NF-YA antibody B. using mock transfected (ctr) and a dominant negative NF-Y (dnNF-YA) transfected SW480 cells **C.** ChIP-qPCR analysis, on SW-480 cells, of CXCR4 promoter that does not contain CCAAT boxes used as unrelated promoter with anti-LMNA/C, LMNA and anti-NF-A antibodies. **D.** ChIP–qPCR analysis, with anti-LaminA/C antibody, of a telomeric region (ITS18-1), a DNA region immediately adjacent to the telomere (Tel-Adj), and a DNA region without an ITS (RPLP0). In all experiments, the ChIP-qPCR results obtained by 3 independent replicate experiments are represented as percentage of input (% Input) on a logarithmic scale, the error bars indicate the standard error. No antibody values were subtracted. The error bars indicate the standard error.

To address this hypothesis we performed ChIP experiments followed by quantitative real time PCR (ChIP-qPCR). Specific primers were used to amplify DNA regions encompassing NF-Y consensus sites on several NF-Y target promoters. Our experiments performed with two different antibodies against lamin A, demonstrate that it associates, although at different extent, with several promoter regions carrying the CCAAT-boxes, such as CCNB2, DHFR, CCNA2, CDK1, CCNB1, CDC25C, TOPO2A and PCNA promoters (Figure 3A). As expected [9], NF-Y binds all these promoter regions (Figure 3B). In contrast, when using primers corresponding to sequence of an unrelated promoter (CXCR4 promoter) that does not contain CCAAT boxes, we did not find any specific lamin A *in vivo* recruitment (Figure 3C). Moreover, we validated the specificity of anti- lamin A/C antibody used in ChIP-qPCR. It has already been demonstrated that lamin A/C specifically associates with sequences named interstitial telomeric sequences (ITSs) [46]. Indeed, we performed ChIP–qPCR analysis in SW-480 cells of a telomeric sequence (ITS18-1), of a sequence in close proximity of the telomere (Tel-Adj), and of a site without an ITS (RPLP0). As shown in Figure 3D, our data confirmed the specificity of lamin A/C enrichment at the ITS region, whereas the association was not observed at the Tel-Adj site and at the RPLP0 site.

To directly assess the role of NF-Y in the recruitment of lamin A to the NF-Y target promoter regions carrying CCAAT boxes, we performed ChIP experiments upon overexpression of dnNF-YA mutant protein in SW480 cells. ChIP-qPCR analysis shows that the overexpression of dnNF-YA induces a decreased recruitment of NF-Y (Figure 3B), and this correlates with a reduction of lamin A binding in five of the eight promoter regions analyzed, such as CCNB2, DHFR, CDC25C, TOPO2A and PCNA, (Figure 3A), although at different extent depending on the analyzed promoter. These data suggest that the binding of lamin A to some promoter regions carrying CCAAT-boxes may be dependent on the binding of NF-Y complex.

Very recently, it has been demonstrated that lamin A/C is able to bind to specific DNA sequences [47]. We investigated the presence of these sequences in the promoter regions analyzed (Supplementary Figure 4A). We found some of them in the close proximity of the CCAAT boxes but, very interestingly, they were particularly enriched in CDK1, CCNA2 and CCNB1 promoters that did not show any modulation of lamin A/C recruitment after dnNF-YA overexpression, thus suggesting that lamin A/C may interact through its direct DNA binding in these promoter regions.

Taken together, our data demonstrate that the binding of lamin A to CCAAT containing promoter regions of several cell cycle genes is largely dependent on the binding of NF-Y complex, and indicate that lamin A binds these promoters through its ability to bind NF-Y.

### Lamin A impacts on NF-Y transcriptional activity

**Figure 4:**
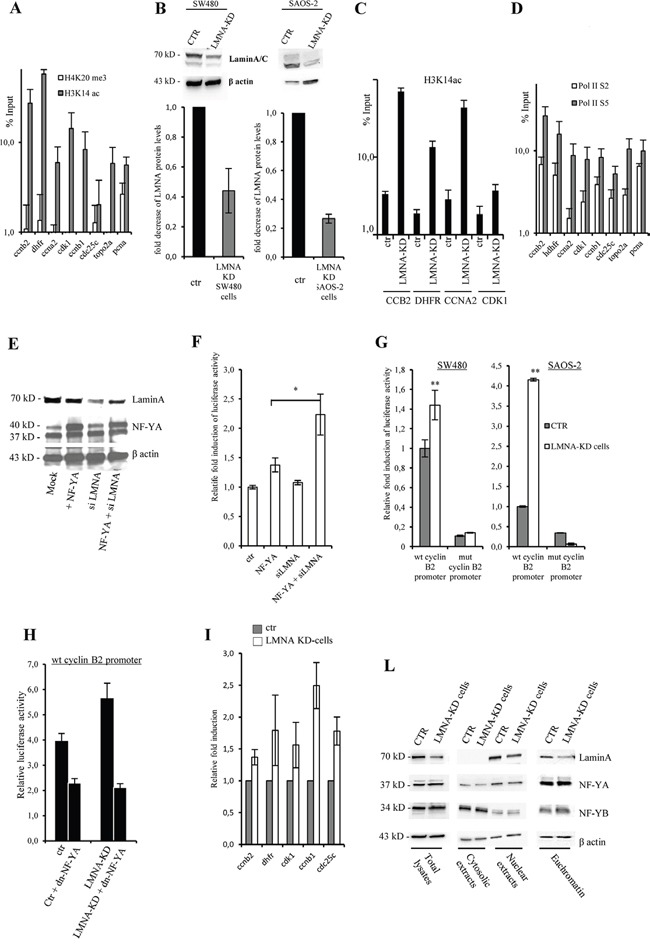
Lamin A is involved in NF-Y transcriptional activity **A.** ChIP-qPCR analysis performed on SW480 using anti-H3K4me3 and H3K14ac antibodies using specific primers to amplify DNA regions encompassing NF-Y consensus sites on CCNB2, DHFR, CCNA2, CDK1, CDC25C, TOPO2A, and PCNA promoters. **B.** Western blotting of total cell lysates from SW-480 and SAOS-2 mock transfected (CTR) or LMNA-KD stable transfected cells (LMNA-KD) with lamin A/C antibody. Bands were quantified by densitometry using UVI-1D quantification module. Histogram shows the quantitative densitometry of lamin A protein (fold over control) normalized over β actin expression, as mean S.E. from three independent experiments. **C.** ChIP-qPCR analysis performed on SW480 mock transfected (CTR) or LMNA-KD stable transfected cells (LMNA-KD), using anti-H3K14ac and specific primers to amplify DNA regions encompassing NF-Y consensus sites on CCNB2, DHFR, CCNA2, CDK1. **D.** ChIP-qPCR analysis performed on SW480 using anti-pol II phospho-ser 3 (Pol IIS2) and pol II phosphor-ser 5 (Pol IIS5) antibodies using specific primers to amplify DNA regions encompassing NF-Y consensus sites on CCNB2, DHFR, CCNA2, CDK1, CDC25C, TOPO2A, and PCNA promoters. The ChIP results obtained by 3 independent replicate experiments are represented as percentage of input (% Input) on a logarithmic scale, the error bars indicate the standard error. No antibody values were subtracted. In (A), (C) and (D) mean of 3 independent replicate experiments are represented as percentage of input (% Input) on a logarithmic scale, the error bars indicate the standard error. **E.** Western blotting showing the expression levels of lamin-A and NF-YA proteins in SW480 cells transiently transfected with cyclin B2 promoter construct driven luciferase gene. Cells were transiently co-transfected with the indicated vectors and used for luciferase assays. β actin was used as loading control. **F.** Luciferase assays performed in SW480 cells described above after 48 hrs post transfection. Promoter activity is expressed as fold change of firefly/Renilla luciferase ratio. Results were obtained by eight independent replicate experiments. **G.** Luciferase assays performed with LMNA knock-down in SW480 cells (LMNA-KD), transfected with the CCNB2 luciferase construct carrying the wild type cyclinB2 promoter (wt cyclinB2 promoter) or cyclin B2 promoter mutated on the 3 CCAAT boxes (mut cyclinB2 promoter). Promoter activity is expressed as fold change of firefly/Renilla luciferase ratio. Results were obtained by 10 independent replicate experiments. The error bars indicate the standard error. **H.** Luciferase assays performed in SW480 cells transfected with cyclin B2 promoter construct driven luciferase gene. Cells were transiently co-transfected with the indicated vectors and luciferase assays were performed after 48 hrs. Promoter activity is expressed as fold change of firefly/Renilla luciferase ratio (n=3). **I.** qPCR analysis of expression levels of the indicated mRNA in LMNA-KD versus mock transfected cells (ctr) (n=3). **L.** Western blotting analysis with the indicated antibodies of whole cell lysate (total lysates), cytosolic and nuclear extracts and euchromatin fraction with the indicated antibodies produced from mock transfected (CTR) or LMNA-KD stable transfected SW480 cells. Statistical significance: *p<0.05. **p<0.01.

To characterize the functional role of the lamin A binding to NF-Y target promoters, we investigated the chromatin structure of the promoter regions bound by lamin A/C and NF-Y. We performed ChIP-qPCR experiments using antibodies against H4K20me3 and H3K14ac, hallmarks of closed and open chromatin structure, respectively. Previous analysis demonstrated an excellent correlation between NF-Y binding and regions enriched for histone modifications associated with active transcription [9]. As expected, we observed an enrichment of H3K14ac on these regions thus demonstrating their open chromatin configuration and transcription-permissive state (Figure 4A).

Thus, we asked whether the depletion of lamin A affects histone modifications and we compared the recruitment of H3K14ac on several NF-Y target promoter regions in cells silenced for lamin A. On this purpose, we produced stable siLMNA SW-480 and SAOS-2 cells (LMNA-KD cells) showing a reduction in the protein level of lamin A of about 60% and 70%, respectively, compared to control cells transfected with the same vector carrying a non-targeting artificial siEmGFP-miRNA (Figure 4B). Results shown in Figure 4C demonstrate an increase of H3K14ac binding in LMNA KD cells, suggesting a specific role of lamin A on regulation of cell cycle gene promoters target of NF-Y activity.

Next, to investigate the transcriptional activity of these promoter regions we performed ChIP-qPCR experiments using antibodies against RNA polymerase II active isoforms. Of note, the transcriptionally active phosphorylated forms of RNA Polymerase II (Ser-2 and Ser-5) are recruited to all the analyzed regions, thus further indicating gene activation (Figure 4D). The same results were obtained by ChIP followed by semiquantitavie PCR, amplifying *CCNB2* promoter as prototype of NF-Y target gene (Supplementary Figure 4B, 4C). Altogether, the results clearly demonstrate that lamin A may interact with NF-YA on chromatin of NF-Y target genes in an open conformation status and actively transcribed.

We confirmed the involvement of lamin A on NF-Y target gene transcription using CCNB2 promoter driven luciferase reporter construct as a sensor of NF-Y activity. We transiently overexpressed this construct in SW-480 cells together with a vector expressing NF-YA and/or a vector that allows the simultaneous expression of an artificial miRNA able to target the mRNA of lamin A, and the EmGFP reporter gene as control (pcDNA6.2-GW/EmGFP-miR-LMNA) [33]. Our data suggest that down-modulation of lamin A, although not complete (Figure 4E), led to a significant increase of CCNB2 promoter activity upon NF-YA overexpression (Figure 4F) thus supporting the hypothesis that lamin A may interfere with NF-Y activity. To validate these data, we used LMNA-KD cells. We observed an increase of CCNB2 promoter activity in LMNA-KD cells (Figure 4G). We also employed a CCNB2 promoter construct carrying 3 mutated CCAAT boxes (mut cyclin B2 promoter). As already shown [4], the basal CCAAT-less promoter activity of the mut cyclin B2 promoter was significantly reduced compared to the wt construct but lamin A modulation did not affect significantly the residual activity (Figure 4G). Since lamin A knockdown increases cylin B2 promoter activity, we asked whether this activity go back down by inhibiting NF-Y activity. To answer this question we overexpressed cyclin B2 promoter driven luciferase reporter construct together with the vector expressing dnNF-YA protein in LMNA-KD and control cells. As expected, dnNF-YA inhibits cyclin B2 promoter and, of note, it is able to rescue its activity upregulation observed in cells lacking lamin A (Figure 4H). These data indicate that lamin A depletion activates cyclin B2 promoter transcription through NFY activation. In agreement with the above results we observed in LMNA-KD cells an increase of the amount of several NF-Y target mRNAs such as CCNB2, DHFR, CCNB1, CDC25C and CDK1 (Figure 4I). As shown in Figure 4L, NF-Y localization on euchromatin was not affected by lamin A silencing. This result suggests that, very likely, the enhanced NF-Y transcriptional activity observed by lamin A silencing depends on a decreased number of lamin A molecules able to associate with NF-Y and inhibits its activity.

**Figure 5:**
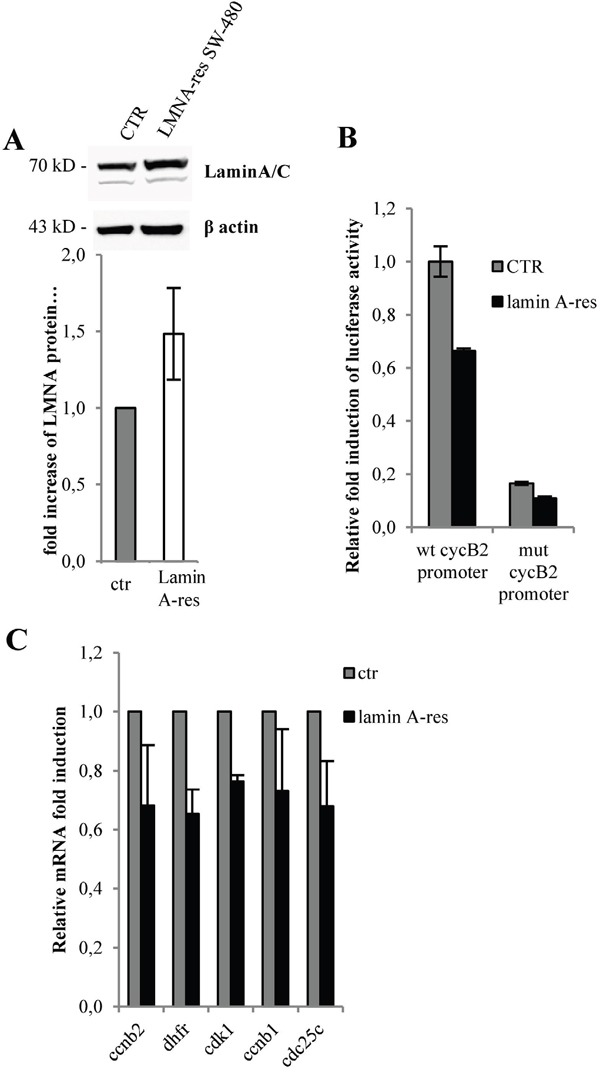
Lamin A inhibits NF-Y-dependent gene expression **A.** Western blot performed with total lysate from mock or stably transfected SW480 cells with a human lamin A expression vector coding for a laminA resistant to siRNAs (lamin A-res). Bands were quantified by densitometry using UVI-1D quantification module. Histogram shows the quantitative densitometry of lamin A protein (fold over control) normalized over β actin expression, as mean S.E. from three independent experiments. **B.** Luciferase assays performed in lamin A-res SW480 cells versus mock cells (CTR) transfected with the CCNB2 promoter luciferase construct (wt cyclin B2 promoter) or cyclin B2 promoter carrying mutated CCAAT boxes (mut cyclin B2 promoter) (n=3). **C.** Expression levels by qPCR of the indicated mRNAs in lamin A-res SW480 cells (lamin A-res) versus mock cells (ctr) (n=3). In all experiments, the error bars indicate the standard error. Statistical significance: *p<0.05, **p<0.01.

Next, we stably transduced in SW-480 cells a human lamin A (lamin A-res) expressing vector resistant to siRNAs [48] (Figure 5A). Lamin A resistant cDNA was designed to contain several silent codon changes at the target sites of the miRNAs, thereby rendering it resistant to the endogenous miRNAs. Consistent with our previous results, expression of lamin A-res led to a reduction of the basal CCNB2 promoter activity of approximately 30% (Figure 5B) and to a decrease of CCNB2, DHFR, CCNB1, CDC25C and CDK1 mRNA levels (Figure 5C). Also in these cells, CCNB2 promoter construct carrying two mutated CCAAT boxes (mut CCNB2 promoter) was not affected by lamin A modulation (Figure 5B).

**Figure 6:**
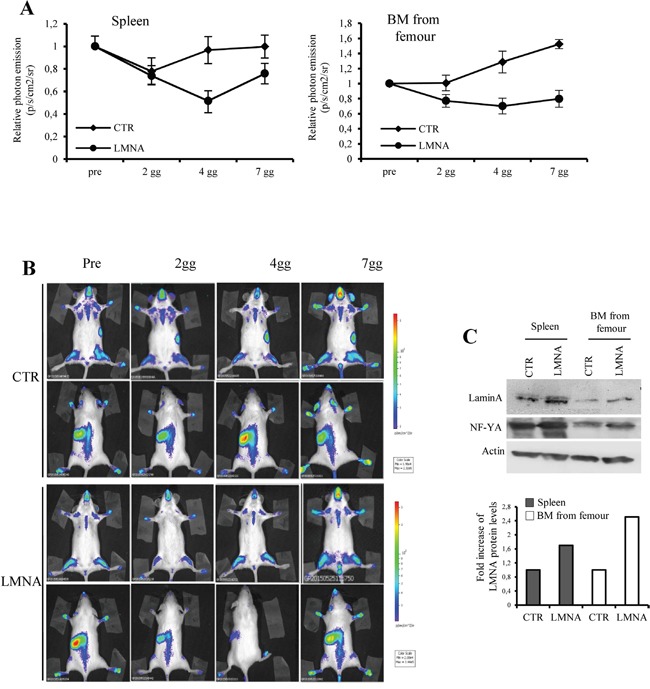
Lamin A impact on NF-Y activity in vivo **A.** Quantification of emitted photon emission from femurs and spleen of MITO-Luc mice was determined before infection (pre), 2, 4, and 7 days after injection (2gg, 4gg, 7gg). The retroviruses pMXIH-V5 (CRT), and pMXIH-human lamin A-res (LMNA) were used for infections. Bioluminescence was expressed as relative p/s/cm2/sr. **B.** Bioluminescence imaging of a representative MITO-Luc mouse before (Pre), after 2, 4, and 7 days (2gg, 4gg and 7gg) after infection. The area used for the photon emission quantification in (A) are indicated (femurs and spleen). The experiments were performed in eight animals. **C.** Western blotting analyses performed with total extract from bone marrow (BM) from long bones and spleen of mice after 7 days post-infection. Densitometry was performed with the ImageJ software (National Institutes of Health), values were normalized to actin and folds quantified with respect to untreated organs (CTR) set to 1.0.

To demonstrate *in vivo* the impact of lamin A on NF-Y transcriptional activity we took advantage of the MITO-Luc mouse model, that we recently developed, carrying a luciferase reporter driven by a promoter strictly dependent on NF-Y allowing us to monitor the NF-Y activity within the entire living organism in a spatiotemporal manner [49]. We demonstrated that intravenous injection of a viral vector expressing dn-YA in MITO-Luc mice an almost complete inhibition of luciferase activity is observed in every body area. Thus, we reasoned that it was possible to overexpress lamin A in our MITO-Luc mice by injecting the viral vector encoding lamin A-res described above [48]. We injected MITO-Luc mice intravenous with lamin A-res viral vector and we followed luciferase activity in the entire animals for several days. We have previously described that in MITO-Luc mice high luciferase activity is exerted in the bone marrow contained in the long bones and in the spleen [49]. Of note, lamin A-res injection results in an inhibition of luciferase activity emitted by these tissues at any tested time (Figure 6A). In these graphs the values coming from the pre injection imaging session are reported as 1 and the other values are relative to this value in each mouse. Representative images of a control and injected mouse are shown in Figure 6B. It is important to notice that the values are relative to the value of mouse is imagined Western blot analysis conducted on extracts from these tissues at 7 days after lamin A-res injection confirmed the overexpression of lamin A in these tissues (Figure 6C). Taken together, our results demonstrate that lamin A impacts on NF-Y transcriptional activity in cell cultures and living animals.

**Figure 7:**
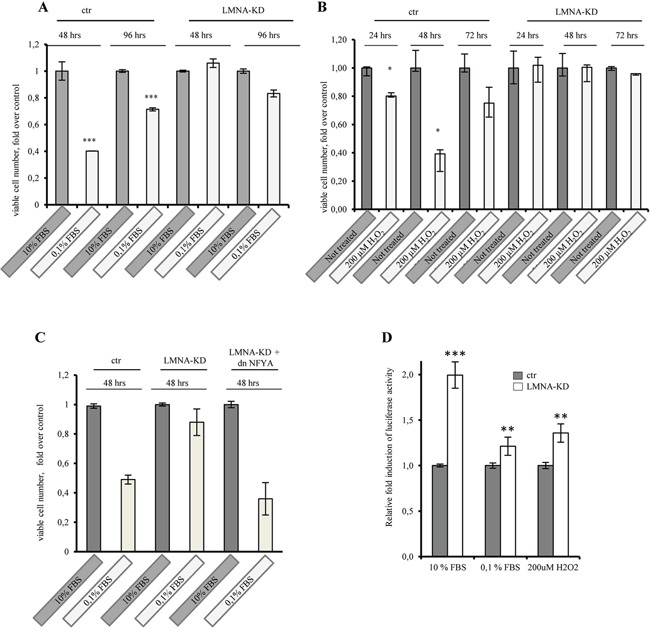
Lamin A knock-down impacts on cell proliferation through NF-Y activity SW480 mock transfected cells (ctr) and SW480 lamin A knock-down (LMNA-KD) cells were grown in 10% serum, 0, 1% serum or in 200 μM hydrogen peroxide (H2O2), viable cell number were counted daily. **A.** The growth of SW480 cells was partially reduced upon growth factor deprivation or **B.** upon oxidative stress conditions compared with control cells while the growth of SW480 LMNA-KD cells was not impaired. **C.** dnNF-YA expression rescue lamin A knock-down-mediate increase in cell proliferation in cells cultured in 0, 1% serum. **D.** Luciferase assays performed in SW480 LMNA knock-down (LMNA-KD) and in mock transfected cells (ctr) transiently transfected with the cyclinB2 luciferase promoter construct. Luciferase activity was normalized by Renilla luciferase, and means of 3 experiments performed in triplicates is presented as fold induction where luciferase in ctr cells is = 1. In all experiments, the error bars indicate the standard error. Statistical significance: *p<0.05, **p<0.01, ***<p< 0,001.

To investigate the role of lamin A in cell cycle progression, we performed time course experiments and compared the ability of SW-480 and SW-480 LMNA-KD cells to grow in 0, 1% serum or after hydrogen peroxide treatment (200μM H_2_O_2_). The growth of SW-480 cells was partially reduced upon growth factor deprivation (Figure 7A) or upon oxidative stress conditions (Figure 7B) compared with control cells, whereas the growth of SW-480 LMNA-KD cells was always not impaired indicating that lamin A knockdown increases cell proliferation. We asked whether NFY inhibition would rescue lamin A knockdown phenotype on cell proliferation. To answer this question we overexpressed dnNFYA in LMNA-KD cells and we observed a decrease of cell proliferation (Figure 7C). This results, together with the higher basal CCNB2 promoter luciferase activity observed both under serum deprivation and oxidative stress conditions in LMNA-KD cells compared with control cells (Figure 7D), suggests that the lamin A knockdown-mediated increase in cell proliferation is actually mediated by NFY activation. Altogether, our data demonstrate lamin A as a novel repressor of NF-Y activity and indicate its role as a suppressor of cell proliferation. Moreover, we hypothesize a role of lamin A as a sensor of cellular stress, such as serum deprivation and oxidative stress.

## DISCUSSION

In the present study, starting with a mass-spectrometry screening, we identified a novel nuclear protein complex formed by lamin A and NF-Y involved in chromatin binding and cell proliferation.

NF-Y is a transcription factor able to binds the common CCAAT element and is generally considered a modulator of genes involved in growth promotion such as cell cycle regulatory genes [7, 9]. Numerous findings highlight that NF-Y has a role in cancer. Although mutations in NF-Y subunits have never been specifically identified in tumours, analysis of global regulatory perturbations across human cancers pointed at NF-Y as one of the transcription factors responsible for oncogenic transcriptional changes [19]. Thus, identification of NF-Y protein partners can help to characterize the mechanism associated with its tumorigenic potential.

Here, we demonstrated that lamin A associates with NF-Y, whereas lamin C is not present in the complex. This result is in accordance with a previous study reporting that lamin A and C are found, at least in part, in different complex within the lamina [31] and thus may participate to different protein complexes. We also provide evidences that lamin A interaction may affects NF-Y binding activity on promoter regions encompassing CCAAT boxes of NF-Y target genes modulating its transcriptional activity.

Although there are some evidences that lamin A binds DNA, directly or through the histone proteins, the role of nucleoplasmic lamin A is not completely understood, so far [35, 50]. Thus, we focused our study on the occurrence of lamin A/NF-Y association in the nucleoplasm compartment and, in particular, on chromatin where NF-Y exerts its role as transcription factor. In our study, we detected lamin A enrichment in promoter regions encompassing CCAAT boxes of several NF-Y target genes. These genes are actively transcribed as demonstrated by histone methylation marks and pol II recruitment. Interestingly, we observed that lamin A recruitment depended on NF-Y binding activity in five of the eight promoter regions analysed, such as CCNB2, DHFR, CDC25C, TOPO2A and PCNA, and at different extent, thus indicating that lamin A may specifically modulate NF-Y regulated promoter regions not only through interaction with NF-Y, but also via other proteins or through direct DNA binding, according with recent evidences described in a recent paper indicating that lamin A/C directly binds to specific motifs in DNA segments [47].

Lamin A has been described to have a negative role on transcription. We clearly demonstrate that lamin A inhibits NF-Y transcriptional activity. These results suggest, therefore, that lamin-promoter interactions, per se, do not have a causative role on gene repression but may be able to modulate transcription in a manner dependent on local chromatin marks.

Numerous studies showed that lamin A can modulate gene expression through different mechanisms, among which the modulation of transcription factor subnuclear localization, the sequestering of transcription factors in inactive complexes, the regulation of their degradation [47, 51–55]. Interestingly, we observed that the NF-Y target CCNB2 promoter activity inversely correlated to lamin A expression and NF-Y transcriptional activity increases in lamin A silenced cells. Of note NF-Y localization on euchromatin was not affected by lamin A silencing (Figure 4F). This result suggests that, very likely, the enhanced NF-Y transcriptional activity observed in lamin A silenced cells depends on a decreased number of lamin A molecules able to associate with NF-Y and inhibit its activity. These results are coherent with previous evidences demonstrating thatdown-regulation of lamin A/C leads to dissociation of lamin A/C from promoters by enhancing transcriptional permissiveness [53]. It has been observed that lamin A interactions often appear to be confined to promoter subregions rather than to entire promoter regions. Our data support a view in which lamin A, through its ability to bind NF-Y, exerts a locus-specific interaction with promoters important for cell cycle regulation and tumor progression.

In our study, an inverse correlation between lamin A and several NF-Y target genes expression level was observed, thus supporting the role of lamin A as regulator of NF-Y transcriptional function. We validated these evidences by *in vivo* imaging involving the use of a genetically engineered mouse model called MITO-Luc (for mitosis-luciferase), in which an NF-Y–dependent promoter drives luciferase expression. Data obtained strongly support the physiological impact of lamin A expression in cell proliferation.

NF-Y is one of the transcription factors responsible for aberrant oncogenic transcription occurring in several cancers [19]. Loss of lamin A expression has been reported in several cancers, and has been correlated with a more aggressive tumorigenic phenotype [56–60]. It is worth to note that cancer cells from solid tumors become metabolically stressed, when nutrients are insufficient within poorly vascularized regions. Metabolic stress results from severe deprivation of oxygen, glutamine and glucose, partly through excessive reactive oxygen species (ROS) production. Oxygen radicals may enhance cell migration rates and consequently increase tumor invasion and metastasis. Interestingly, our data obtained treating cancer cells under low nutrient and oxidative stress conditions indicate that loss of lamin A in cancer cells is associated with an increase NF-Y translational activity and with an increase of cellular proliferation ability compared with control cells. These data suggest that changes in lamin A expression could modulate NF-Y activity and, in particular, lamin A lost may correlate with an increase NF-Y oncogenic transcriptional potential.

Further exploration to uncover the molecular mechanism(s) by which NF-Y/ lamin A complex acts as crucial regulator in diverse cellular processes and, in particular, in cancer could be important to improve and potentially provide new clues into new therapeutic approaches for cancer treatment.

## MATERIALS AND METHODS

### Immunoprecipitation assay and mass spectrometry

Lysates (2 mg/sample) were precleared with Protein-G Agarose (Pierce) and incubated 4h at 4°C with an anti-NF-YA polyclonal antibody-protein-G complexes (50ul proteinG + 5ug antibody/sample) previously crosslinked by DMP (Sigma). We used two negative controls : 50ul of anti-NF-YA polyclonal antibody-protein-G complexes immunoprecipitated without extract (ctr ab), 2 mg of cell extract immunoprecipitated with 50ul of protein-G without the antibody (ctr cell extract).

Immunoprecipitated proteins were resolved on a 12%T- 3.3%C SDS–PAGE separating gel (1+18+18 mm), revealed by Sypro Ruby staining and visualized using a Typhoon 9200 laser scanner (GE Healthcare). Proteins were excised from the gel using Investigator ProPic spot picker (Genomic Solutions) and transferred to small tubes (0.2 ml). Protein-containing gel pieces were destained with 50 ml of 0.1M ammonium bicarbonate (5 min at RT). After two washes with 50% acetonitrile/0.05M ammonium bicarbonate, gel plugs were shrunk by addition of 100% acetonitrile. The dried gel pieces were re-swollen with 4.5 ng/ml trypsin in 50mM ammonium bicarbonate and digested overnight at 378C. Peptides were concentrated with ZipTipmC18 pipette tips (Millipore). Coelution was performed directly onto a MALDI target with 1 ml of a-cyano-4-hydroxycinnamic acid matrix (5 mg/ml in 50% acetonitrile, 0.1% TFA). Proteins were identified by Matrix Assisted Laser-Desorption Ionization (MALDI)-MS and MALDI-MS/MS (4700 Proteomics Analyzer; Applied Biosystems). Data were acquired in positive MS reflector mode. Five peptides (ABI4700 Calibration Mixture; Applied Biosystems) were used as calibration standards. Mass spectra were obtained from each sample by 30 sub-spectra accumulation (50 laser shots each) in a 750 to 4000. mass range. Five signal-to-noise best peaks of each spectrum were selected for MS/MS analysis. For MS/MS spectra, the collision energy was 1 keV and the collision gas was air. The interpretation of both the MS and MS/MS data was carried out by using the GPS Explorer software (Version 1.1, Applied Biosystems), which acts as an interface between the Oracle database containing raw spectra and a local copy of the MASCOT search engine (Version 1.8). Peptide mass fingerprints obtained from MS analysis were used for protein identification in the NCBI non redundant database. All peptides mass values were considered monoisotopic and mass tolerance was set at 50 p.p.m. One missed cleavage site was allowed, cysteines were considered carboamidomethylated, methionine was 0assumed to be partially oxidized and serine, threonine and tyrosine partially phosphorylated. Mascot (Matrix Science) scores >61 were considered significant (P<0.005). For MS/MS analysis, all peaks with a signal-to-noise ratio >5 were searched against the NCBI database using the same modifications as the MS database, with a fragment tolerance <0.3 Da.

### Cell lines

Human breast cancer cell lines MCF-7 and SKB-R3, human colorectal carcinoma cell lines HCT-116 and SW-480, human osteosarcoma with osteoblastic properties cells Saos-2, human primary fibroblasts (HF), and human cervical adenocarcinoma HeLa cells were all cultured in Dulbecco's modified Eagle's medium (DMEM, Gibco BRL) supplemented with 10% fetal calf serum and antibiotics in a humidified 5% CO_2_ atmosphere.

### Total lysates extraction

Cells, harvested in cold phosphate-buffered saline, were extracted for 30 min at 4°C in lysis buffer (50 mM Tris-HCl, pH 7.4, 150 mM NaCl, 1% NP-40) containing 1 mM DTT, protease inhibitor cocktail (Roche) and phosphatase inhibitors (50 mM NaF, 0.2 mM Na3VO4). After centrifugation, supernatants were collected as the total protein extract and stored at -80°C. Protein concentrations were measured using the Bradford-type protein assay (Bio-Rad).

### Lamins solubilization

Cells were resuspended in hypotonic buffer (10 mM Hepes pH 7.9, 1.5 mM MgCl2, 10 mM KCl, 0.5% NP-40, 0.5 mM DTT, and protease inhibitors) and nuclei separated by centrifugation. Nuclei were then incubated 30 min in moderate-salt buffer buffer (300 mM KCl, 2% Triton X-100, 10% sucrose, 20 mM MES-KOH pH 6.0, 2 mM EDTA, 1 mM DTT) and centrifuged (3, 300g, 15 min, 4°C) to obtain a lamin-enriched pellet. This pellet was incubated on ice 30 min in high pH/high detergent buffer (300 mM KCl, 2% Triton X-100, 20 mM Tris-HCl pH 9.0, 2 mM EDTA, 1 mM DTT) followed by centrifugation (6, 000g, 20 min) to yield a supernatant of solubilized lamins (soluble lamins) and an insoluble pellet.

### Chromatin extraction

Cells were lysed by mechanical homogenization in isotonic- sucrose based buffer (15 mM Tris-HCl pH 7.5, 15 mM NaCl, 60 mM KCl, 5 mM MgCl2, 1 mM CaCl2, 250 mM Sucrose, 0.3% NP-40, and protease inhibitors) followed by centrifugation (5 min, 1,300 g, 4°C). The pellet, containing the nuclei, was resuspended and lysed in lysis buffer (20 mM Hepes pH 7.9, 1.5 mM MgCl2, 150 mM KOAc, 3 mM EDTA, 10% glycerol, 1 mM DTT, 0.1% Nonidet P-40 and protease inhibitors) and centrifuged (5 min, 1,700 g, 4°C). The supernatant corresponds to the nucleoplasmic fraction and the pellet to the chromatin fraction.

### Euchromatin isolation

Chromatin fraction, obtained as described above, was digested im MNase buffer (20 mM Tris pH 7.5, 15 mM NaCl, 1 mM CaCl2) with Micrococcal nuclease to a final concentration of 1.2 units/mL for 5 minutes, and the reaction was quenched by 1 mM EGTA on ice for 10 minutes. The sample was centrifuged at 1,000× g for 5 minutes at 4°C to generate the supernatant, corresponding to the euchromatin fraction. The insoluble chromatin pellet was resuspended in 15 mM Tris, pH = 7.5, 0.5% SDS. Extraction of DNA

DNA was isolated from chromatin samples by extraction with an equal volume of phenol/chloroform (1:1), extraction with an equal volume of chloroform, ethanol-precipitated and then resuspended in water and loaded onto a 2% agarose gel.

### Immunoprecipitation and immunoblotting

For immunoprecipitation experiments, lysates were clarified and immunoprecipitated at 4°C overnight in lysis buffer by adding protein G-agarose after 2 h of incubation with 2 μg of antibody. Proteins were resolved by SDS-PAGE and electro transferred to nitrocellulose. Each membrane was blocked with 5% non-fat dry milk in Tris buffered saline-Tween-20 (TBST) for 1 h at room temperature and subsequently incubated with primary antibody for 16 h at 4°C. The following antibodies were used: anti-NF-YA polyclonal (Santa Cruz), anti NF-YA monoclonal (Santa Cruz) and anti-lamin A (Santa Cruz) and anti-lamin A/C (Santa Cruz), anti-β actin (sigma-aldrich). Immunoreactivity was detected by sequential incubation with HRP-conjugated secondary antibody.

For tissue total exstract preparation, after tissue dissection, the organs were immediately frozen on dry ice. Bone marrow was flushed out from the femur and tibia, as previously described [17]. Tissue extracts were prepared by homogenization in a 100 mM KPO4 lysis buffer (pH 7.8) containing 1 mM dithiothreitol, 4 mM ethylene glycol tetraacetic acid, 4 mM EDTA, and 0.7 mM phenylmethylsulfonyl fluoride.

### Double immunofluorescence labeling and confocal microscopy analysis

Cells were fixed with 2% formaldehyde, permeated with 0,05% Triton X-100, blocked 1h with 5% BSA, and subjected to staining using anti-lamin A (Santa Cruz), anti-NF-YA monoclonal (Santa Cruz), Cy3-conjugated donkey anti-mouse and Cy2-conjugated donkey anti-rabbit (Jackson Immuno Research Laboratories). Slides were analyzed within 24 h. As control, single immunofluorescence labeling for each antibody, and immunofluorescence labeling without primary antibody was performed (data not shown). All experiments were performed several times with similar results. Images were recorded by using a Zeiss LSM 510 Meta confocal laser scanning microscope equipped with a 20X and 60X/1.23 NA oil immersion objective. As laser (488 and 514 nm), and HeNe laser (543 nm) were used to excite the fluorophores. The LSM 510 R. 3.2 META (Zeiss) image analysis software was used.

### Plasmids and transfections

Plasmids used in transfections were as follows: NF-YA and empty vector [1], wt cyclin B2 promoter (B2-Luci) and mut cyclin B2 promoter (mutant Y1, 2m-luci) constructs [4], pMXIH-V5 and PMXIH-lamin A res [48], pcDNA6.2-GW and EmGFP-miR-LMNA (Invitrogen).

Cells were transfected with Lipofectamine LTX and Plus reagent (Invitrogen) following the manufacturer's instructions.

Stably transfected cells were selected for by culturing the cells in the presence of blasticidin at10 μg/mL (Invitrogen). The transfected cells were screened using western blotting and real-time RT-PCR assays to determine the levels of lamin A expression and the miRNA expression was monitored by checking the simultaneous coexpression of the EmGFP reporter gene by fluorescence microscopy. For reporter assay luciferase activity was measured using the dual luciferase assay system (Promega) according to the instructions of the manufacturers. All transfections were done as cotransfections with a CMV-driven plasmid expressing Renilla luciferase as internal control to standardize transfection efficiencies.

### ChIP assay

1% formaldehyde was added directly to the cells and incubated at 22 °C for 10 min. The reaction was stopped adding 0.125 m glycine. Then, the cells were rinsed with cold 1× PBS, incubated with 0.2× trypsin-EDTA in 1× PBS, and scraped. cells were centrifuged, washed in cold 1× PBS plus 0.5 mm PMSF and resuspended in lysis buffer (5 mm piperazine N, N bis zethone sulfonic acid (pH 8.85) mm KCl, 0.5% Nonidet P-40). Next, nuclei were solicited in the sonication buffer (0.1% SDS, 10 mm EDTA, 50 mm Tris-HCl (pH 8), 0.5% deoxycholic acid) for 10 min by using a microultrasonic cell disruptor. The chromatin was sheared to an average size of 500 base pairs, and immunoprecipitation was performed with protein G-agarose (KPL). The chromatin solution was precleared by adding protein G for 1 h at 4 °C and incubated at 4 °C overnight with 4 μg of antibody or non-specific immunoglobulins (IgGs, Santa Cruz Biotechnology) as negative control. Input was collected from a control sample supernatant (not immunoprecipitated antibody). Immunoprecipitates were recovered by incubation for 2 h at 4 °C with protein G-agarose precleared previously in immunoprecipitation buffer (1 μg/μl bovine serum albumin, 1 μg/μl salmon testis DNA, protease inhibitors, and PMSF). Reversal of formaldehyde cross-linking, RNase A, and proteinase K treatments were performed. DNA was phenol-extracted, ethanol-precipitated, and analyzed by PCR. DNA representing 0.005–0.01% of the total chromatin sample (input) or 1–10% of the immunoprecipitates was amplified using specific primers indicated in Table 1. The following antibodies were used: anti-Pol II phospho ser 3 (Upstate), anti Pol II phospho ser 5 (Upstate), anti-H3K14ac (Abcam), anti-H4K20me3 (Abcam), anti-NF-YA polyclonal (Santa Cruz), anti NF-YA monoclonal (Santa Cruz) and anti-lamin A (Santa Cruz) and anti-lamin A/C (Santa Cruz). PCR analysis was performed with HOT-MASTER Taq (Eppendorf). Quantitative PCR (qPCR) was performed using SYBR Green (Applied Biosystems) on an ABI Prism 7500 apparatus (Applied Biosystems). Primers used are listed in Table 1.

**Table 1:** PCR primers for ChIP assay

*CCNB2* FWD	ACCGGCTGT TGTGACAATCA
*CCNB2* REV	GGCCAACACAAGATGCACTCT
*DHFR* FWD	CTGGAGACCTAAGGGCAGCTT
*DHFR* REV	TTGGTGGTCGAAGAGTTTTACTGA
*CCNA2* FWD	GCCCCAGCCAGTTTGTTTC
*CCNA2* REV	GGCGAGTGAAGGGTAAACCA
*CDK1* FWD	CGTAGCTGGGCTCTGATTGG
*CDK1* REV	CAAACTCACCGCGCTAAAGG
*CCNB1* FWD	GCCCTGGAAACGCATTCT C
*CCNB1* REV	CCTCCTTATTGGCCTGTTCGT
*CDC25C* FWD	GCTGGTGGGCCAAACACT A
*CDC25C* REV	TGTGCTTGCTCTGGAAATGG
*TOPO 2A* FWD	TGGCCAGATTCCCTGTCAAT
*TOPO2A* REV	AGGTTAGGGAGGCGGGACTA
*PCNA* FWD	CACATATGCCCGGACTTGTTC
*PCNA* REV	CAGGTCTCCCCGCCTCTT
*CXCR4* FWD	AGTGGTTTGACCTCCCCTTT
*CXCR4* REV	ACTTGCACCTGCCAGTCTTC
*ITS18-1* FWD	TCCATGTGGTCTCTCTGTCTGGACT
*ITS18-1* REV	GACGGGCCAGACTGTTGCAT
*Tel-Adj* FWD	CCCCTTGCCTTGGGAGAA
*Tel-Adj* REV	GAAAGCAAAAGCCCCTCTGA
*RPLP0* FWD	GCGCCCATCTAACTAGCACA
*RPLP0* REV	TCGCGACCCTACTTAAAGGC

### RNA extraction and RT-PCR

Total RNA was extracted using the Trizol reagent (Gibco BRL) following the manufacturer's instructions. The first-strand cDNA was synthesized according to the instructions for the M-MLV RT kit (Invitrogen). Quantitative PCR (qPCR) was performed using SYBR Green (Applied Biosystems) on an ABI Prism 7500 apparatus (Applied Biosystems). mRNA expression was normalized for β-actin levels. Primers used are listed in Table 2. Relative mRNA expression was calculated using the comparative Ct method (2−ΔΔCt).

**Table 2:** PCR primers for mRNA quantification

CDK1 FWD	GCGGAATAATAAGCCGGGATC
CDK1 REV	CCCTTATACACAACTCCATAGGT
CDC25C FWD	TCCTGGAGAGAGACACTTCC
CDC25C REV	CAACGTTTTGGGGTTCCTCC
CCNB1 FWD	TGCAGAAGATGGAGCTGATC
CCNB1 REV	GTGACTTCCCGACCCAGTAG
CCNB2 FWD	GCACATGGCCAAGAATGTGGTG
CCNB2 REV	TCAGTGGGGAGGCAAGGTCTT
DHFR FWD	AAACTGCATCGTCGCTGTGTC
DHFR REV	ACCCATAATCACCAGATTCTGT
LMNA FWD	GGACAATCTGGTCACCCGC
LMNA REV	TGGCAGGTCCCAGATTACATG
ACTIN FWD	GGACTTCGAGCAAGAGATGG
ACTIN REV	AGCACTGTGTTGGCGTACAG

### Retroviral infection

Phoenix-ampho cells (American Type Culture Collection) were transfected with pMXIH-V5 or pMXIH-V5 Lamin A. The supernatant medium containing the emerging retrovirus was collected 48 and 72 h after transfection. The supernatants were pooled and the retroviral particles were concentrated by ultracentrifugation at 22.000 rpm for 2 h, resuspended in cold PBS and stored at -80°C. The viral titer was determined infecting NIH3T3 murine fibroblasts at different serial dilutions. The retroviral vectors were injected into the tail vein in adult mice with 5.25x10^2/mouse.

### *In vivo* BLI and experimental animals

MITO_Luc C57Bl/6j [49] were interbred with FVB mice to obtain FVB MITO-Luc mice were maintained in the heterozygous state. After genomic DNA extraction of tail biopsies, the positive founder animals were identified by PCR using the following primers specific for the transgene: oligonucleotide up: 5′-TGTAGACAAGGAAACAACAAAGCCTGGTGGCC; and oligonucleotide down: 5′-GGCGTCTTCCATTTTACCAACAGTACCGG. Light emission was detected using the IVIS Lumina II CCD camera system and analyzed with the Living Image 2.20 software package (Caliper Life Sciences). Mice were anesthetized and 150 mg/kg or 75 mg/kg of D-luciferin were injected IP. Ten minutes later, quantification of light emission was performed in photons/second and visualized in a pseudo-color scaling. Time exposure ranged from 1 to 5 minutes depending on light intensity.

All animal studies were approved by the Institutional Animal Care of the Regina Elena National Cancer Institute and by the Government Committee of National Minister of Health and were conducted according with EU Directive 2010/63/EU for any-animal experiments http://ec.europa.eu/environment/chemicals/labanimals/legislation en.htm.

## SUPPLEMENTARY MATERIALS FIGURES AND TABLES




