# Supplementary material for: The laminA/NF-Y protein complex reveals an unknown transcriptional mechanism on cell proliferation
Source: Oncotarget. 2016 Oct 26;8(2):2628–46. doi: 10.18632/oncotarget.12914 (PMC5356829; doi:10.18632/oncotarget.12914)
Supplement: Supplementary file 1 [file oncotarget-08-2628-s001.pdf]

The laminA/NF-Y protein complex reveals an unknown transcriptional mechanism on cell proliferation

SUPPLEMENTARY TABLE AND FIGURES

Supplementary Table S1: Mass spectrometry (MS) identification of LMNA, HS7C and EF-Tu in NF-YA immunoprecipitated sample. The peptide list resulting from MS analysis of NF-YA immunoprecipitation is reported.

See Supplementary File 1

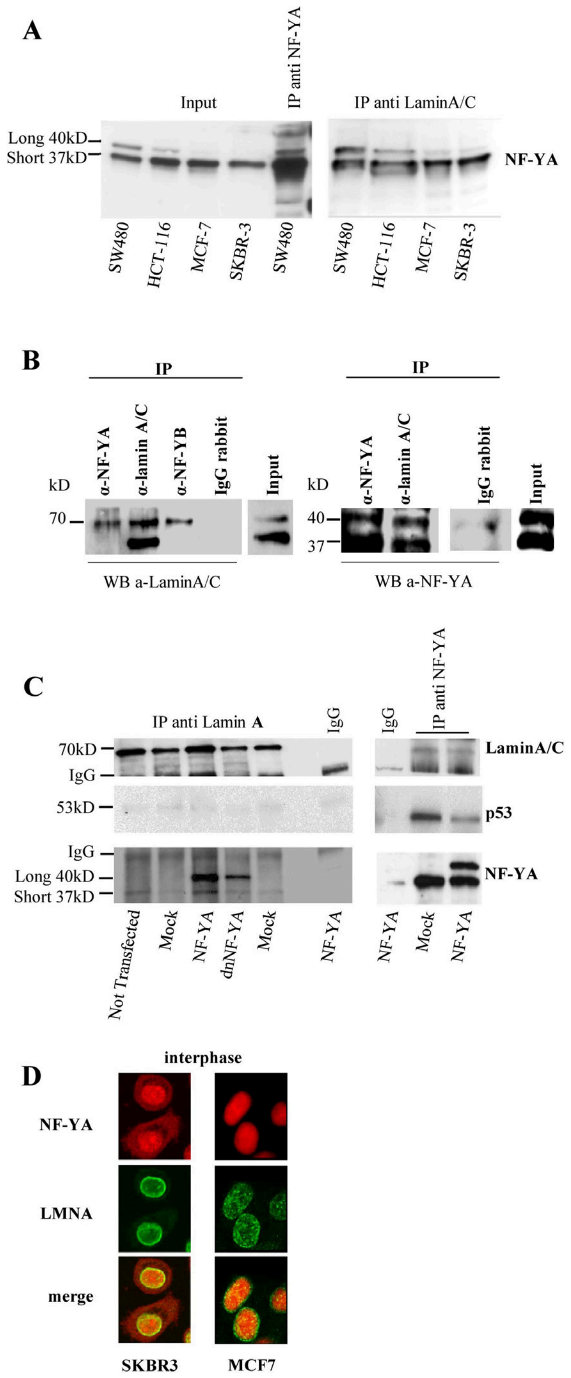

Supplementary Figure S1: Lamin A/NF-Y colocalization. A. Whole cell lysates from several cell lines were immunoprecipitated with an antibody against lamin A/C, and western blotting was performed with an antibody to NF-YA (polyclonal). 1/20 of whole cell extract used in the immunoprecipitations was loaded (input). As negative control, were used SW480 cell lysates immunoprecipitated with an anti-IgG antibody. **B.** Immunoprecipitation experiments using whole cell lystes from SKBR-3 cells performed with antibodies specific to the indicated proteins followed by western blotting with anti-lamin A/C or NF-YA antibody. IgG immunoprecipitate was used as control (IgG). **C.** Immunoprecipitation experiments were performed with whole cell lystes produced from SW480 cells transiently transfected with NF-YA (NF-YA) or a dominant negative mutant of NF-YA subunit (dnNF-YA), or with the empty vector (mock), using antibodies against LaminA and NF-YA. **D.** Confocal analysis of interphase associated localization of NF-YA and lamin A in SK-BR3 and MCF-7 cells. Colocalization (yellow) of endogenous NF-YA (red) with lamin A (green) was analyzed by indirect immunofluorescence combined with Confocal Scanning Laser Microscopy. The images have been collected with a 60x oil objective.

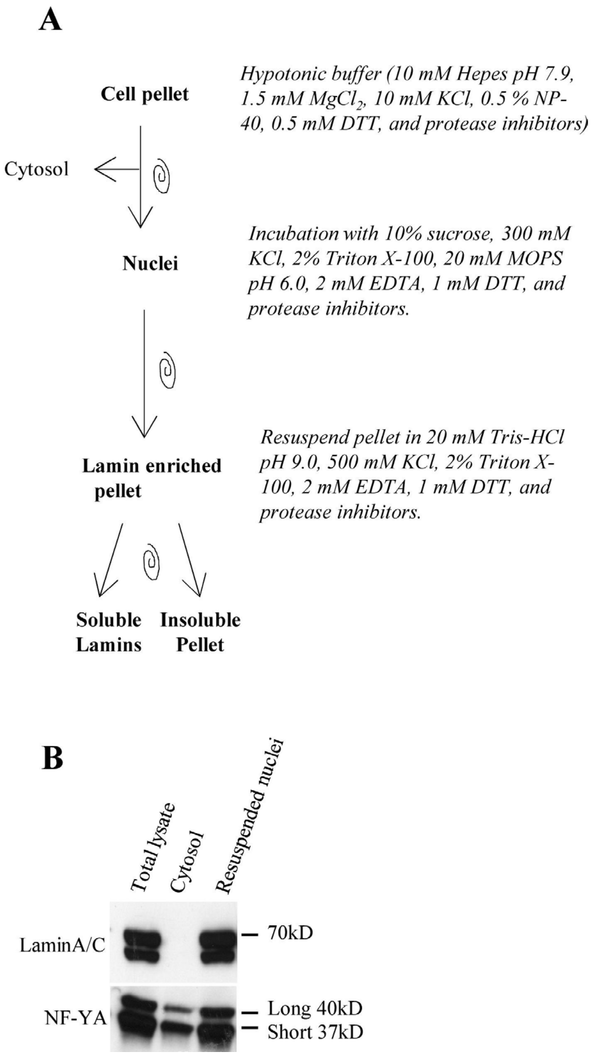

Supplementary Figure S2: NF-Y localization in lamin enriched nuclear fraction. A. Diagrammatic representation of a procedure for lamin solubilization by high-salt and high-detergent buffer. **B.** Western blotting with anti-LMNA and anti-NF-YA antibody of whole cell lysates (total lysate), cytosolic and nuclear (resuspended nuclei) fractions produced as described above obtained from SW480 cells.

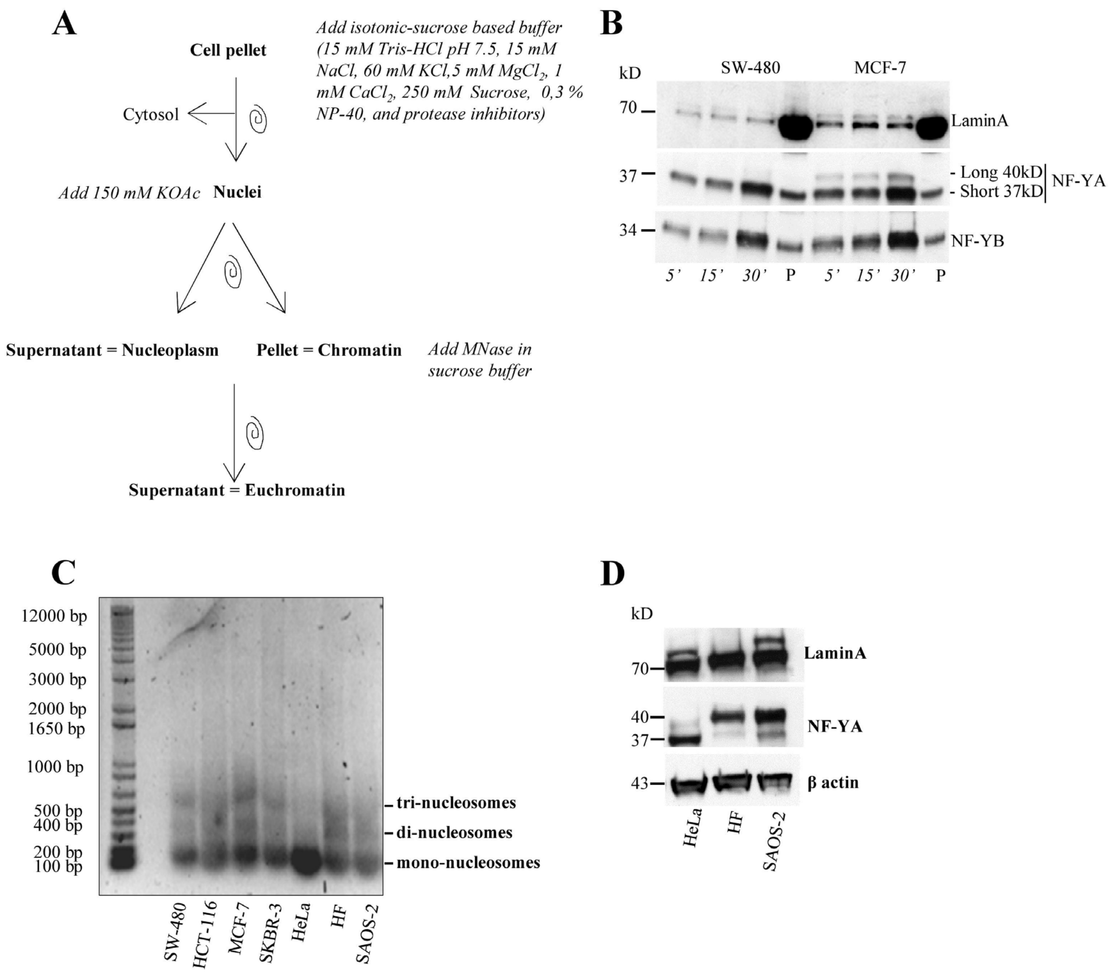

Supplementary Figure S3: lamin A localizes in euchromatin fraction A. Chromatin and euchromatin purification diagram. B. Western blotting analysis with the indicated antibodies of euchromatin and heterochromatin (P) fractions obtained from a time course of MNase digestion on chromatin from SW480 and MCF7 cells produced as described above. The insoluble chromatin pellet (P) was isolated after 30 minutes by centrifugation (1,700× g for 5 minutes at 4°C) and then resuspended in 15 mM Tris, pH = 7.5, 0.5% SDS. Euchromatin fractions were collected after 5, 15 and 30 minutes of MNase digestion. **C.** Euchromatin fractions obtained from the indicated cell lines run on 2% gel agarose collected after 5 minutes of Mnase digestion. Euchromatin fraction is composed mostly of mononucleosomes running at approximately 150 bp. **D.** Western blotting analysis with anti-LaminA/C and NF-YA antibodies of euchromatin fractions produced as described above obtained from human fibroblasts (HF), HeLa and SAOS-2 cells after 5 minutes of digestion.

A

PCNA PROMOTER

AATGTATGCTCTAGGGGGCGGGCCTCGCGGGGAGCATGGACACGATTGGCCCTAAAGTCTTCCCCGC**AAGGCC**GTGGGCTGGACAGC
TGGTGACGTCGC

CDK1 PROMOTER

CACAATATCACTTTTTTGATGAGCTATTTTGAGTATAATAAATTTGAACTGTGCCAATGCTGGGAGAAAAAATTT**AAAAGAA**GAACGG
GCGAACAGTAGCTTCCTGCTCCGCTGACTAGAAACAGTAGGACGACACTCTCCCGACTGGAGGAGAGCGCTTGCGCTCGCACTCAGTT
GCGCCCGCCCTCCTGCTTTTTCTCTAGCC**GCCCTT**TCCTC**TTTCTTTC**GCGCTCTAGCCACC**CGGGAAGGC**CTGCCCAGCGTAGCTGG
CTCTGATTGGCTGCTTTGAAAGTCTACGGGCTACCCGATTGGTGAATCCGGG**GCCCTT**TAGCGCGGTGAGTTTGAAACTGCTC

CCNB1 PROMOTER

CTGTGGCCGCGCCTCTGTCACCTTCCA**AAGGCC**ACTAGGCCTTTCCTGAGCTGGCATTGGCAACGCACACTCTTGCCCGGCTAACCTTT
CAGGTGGGCGGCGCACTGGCTTCACTGCTCTCCAGGTG**GCCGCTGC**AGCTGCCCGAGAGCGCAGGCGCAGAGGCAGACCACGTGAGA
GCCTGGCCA**GGCCTT**CCGGCCTAGCCTCACTGTGGCCCCGCCCCTCTCGAACGCCTTCGCGCGATCGCCCTGGAAACGCATTCTCTGCC
ACCGGCAGCCGCCAATGGGAAGGGAGTGAGTGCCACGAACAGGCCAATAAGGAGGGAGCAGTGCGGGGTTTAAATCTGAGGCTA

CCNA2 PROMOTER

TGCGACCCTGTCGCCTTGAATGACGTC**AAGGCC**GCGAGCGCTTTCATTGGTCCATTTCAATAGTCGCGGGATACTTGAACTGCAAGAA
CA**GCCGCCGC**TCCG

CCNB2 PROMOTER

TTGTGACAATCAAACGTGTC**TAAGAAA**ATTCAGCCAATGAGAGTGCGAGAGTGCATCTTGTGTTGGCCAATGAGAACAGCGACCCGTG
CGCAGGGCCGCCCAATGGGGCGCAAGCGACGCGGTATTTGAATCCTGGAACAAGGCTACAGCGTCGAAGATCCCCAGCGCTGCGGGC
CG

DHFR PROMOTER

GCTGTTGCAGTCTTAAAAACTGGAGACCTAAGGGCAGC**TTTCTTC**TAGTCACCCAATCCAGCACTTTTTTAA

B

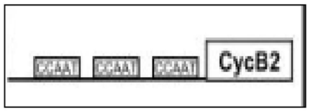

C

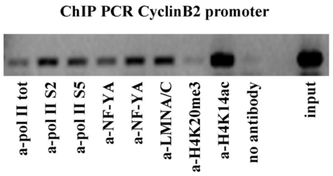

Supplementary Figure S4: lamin A binding activity on *CCNB2* promoter. A. Analysis of the specific motifs in the DNA segments binding with lamin A/C (CVGGAA, GCCGCYGC, and DAAGAAA) identified by Qi YX et al. (47) in proximity of the CCAAT boxes located in promoter regions of *PCNA*, *CDK2*, *CCNB1*, *CCNA2*, *CCNB2,* and *DHFR*. **B.** Schematic representation of the CCAAT boxes on cyclin B2 promoter amplified in ChIP analysis. **C.** Representative agarose gel of ChIP-PCR experiment performed using SW480 cells with the indicated antibodies. Sample without antibody (no antibody) was used as control.
