# Supplementary material for: The laminA/NF-Y protein complex reveals an unknown transcriptional mechanism on cell proliferation
Source: Oncotarget. 2016 Oct 26;8(2):2628–46. doi: 10.18632/oncotarget.12914 (PMC5356829; doi:10.18632/oncotarget.12914)
Supplement: Supplementary file 2 [file oncotarget-08-2628-s002.docx]

**Supplementary Table 1: Mass spectrometry (MS) identification of LMNA, HS7C and EF-Tu in NF-YA immunoprecipitated sample.**

The peptide list resulting from MS analysis of NF-YA immunoprecipitation is reported.

**Protein name Accession No. Protein Protein Total ion Total ion Protein MW Protein Pep. MS Ion**

**Score Score C. Score C.I. % PI Count Intensity**

**1%**

(**P02545) Lamin A/C (70 kDa lamin) LAMA_HUMAN 283 100 185 100 74379.8 6.57 26 2396235**

| **Peptide Information** | |  | | | | | | | | | | |
| --- | --- | --- | --- | --- | --- | --- | --- | --- | --- | --- | --- | --- |
| **Calc. Mass** | **Obsrv. Mass** | | **± da** | **± ppm** | **Start**  **Seq.** | **End**  **Seq.** | **Sequence** | **Ion**  **Score** | **C. I. %** | **Modification** | **Rank** | **Result Type** |

| 807.4359 | 807.4188 | -0.0171 | -21 | 109 | 114 | VREEFK |  | Mascot |
| --- | --- | --- | --- | --- | --- | --- | --- | --- |
| 849.4828 | 849.4839 | 0.0011 | 1 | 42 | 48 | LAVYIDR |  | Mascot |
| 919.438 | 919.4397 | 0.0017 | 2 | 428 | 435 | SSFSQHAR |  | Mascot |
| 972.5109 | 972.5134 | 0.0025 | 3 | 389 | 397 | LSPSPTSQR |  | Mascot |
| 1023.5105 | 1023.5118 | 0.0013 | 1 | 209 | 216 | NIYSEELR |  | Mascot |
| 1028.5735 | 1028.5724 | -0.0011 | -1 | 241 | 249 | LADALQELR |  | Mascot |
| 1089.5535 | 1089.5496 | -0.0039 | -4 | 51 | 60 | SLETENAGLR |  | Mascot |
| 1165.5483 | 1165.5574 | 0.0091 | 8 | 79 | 89 | AAYEAELGDAR |  | Mascot |
| 1182.6113 | 1182.6053 | -0.006 | -5 | 157 | 166 | TLEGELHDLR |  | Mascot |
| 1187.6378 | 1187.6322 | -0.0056 | -5 | 320 | 329 | LRDLEDSLAR |  | Mascot |
| 1243.6641 | 1243.6644 | 0.0003 | 0 | 379 | 388 | LLEGEEERLR |  | Mascot |
| 1293.6433 | 1293.6331 | -0.0102 | -8 | 79 | 90 | AAYEAELGDARK |  | Mascot |
| 1347.6725 | 1347.6649 | -0.0076 | -6 | 367 | 377 | LALDMEIHAYR | Oxidation (M)[5] | Mascot |
| 1363.6172 | 1363.6124 | -0.0048 | -4 | 516 | 527 | AQNTWGCGNSLR | Carbamidomethyl (C)[7] | Mascot |
| 1381.6958 | 1381.6793 | -0.0165 | -12 | 209 | 219 | NIYSEELRETK |  | Mascot |
| 1502.7234 | 1502.7157 | -0.0077 | -5 | 250 | 261 | AQHEDQVEQYKK |  | Mascot |
| 1525.7567 | 1525.7565 | -0.0002 | 0 | 197 | 208 | LQTMKEELDFQK | Oxidation (M)[4] | Mascot |
| 1566.7506 | 1566.7427 | -0.0079 | -5 | 628 | 644 | SVGGSGGGSFGDNLVTR |  | Mascot |

| 1605.8118 | 1605.8057 | -0.0061 | -4 | 440 | 453 | VAVEEVDEEGKFVR | 111 | 100 |  | Mascot |
| --- | --- | --- | --- | --- | --- | --- | --- | --- | --- | --- |
| 1605.8118 | 1605.8057 | -0.0061 | -4 | 440 | 453 | VAVEEVDEEGKFVR |  |  |  | Mascot |
| 1629.8079 | 1629.7994 | -0.0085 | -5 | 29 | 41 | LQEKEDLQELNDR |  |  |  | Mascot |
| 1665.8918 | 1665.8783 | -0.0135 | -8 | 157 | 171 | TLEGELHDLRGQVAK |  |  |  | Mascot |
| 1699.9701 | 1699.9586 | -0.0115 | -7 | 297 | 311 | IRIDSLSAQLSQLQK | 73 | 100 |  | Mascot |
| 1699.9701 | 1699.9586 | -0.0115 | -7 | 297 | 311 | IRIDSLSAQLSQLQK |  |  |  | Mascot |
| 1752.8623 | 1752.8528 | -0.0095 | -5 | 281 | 296 | NSNLVGAAHEELQQSR |  |  |  | Mascot |
| 1761.9229 | 1761.8794 | -0.0435 | -25 | 63 | 78 | ITESEEVVSREVSGIK |  |  |  | Mascot |
| 1909.9211 | 1909.9155 | -0.0056 | -3 | 352 | 366 | MQQQLDEYQELLDIK |  |  | Oxidation (M)[1] | Mascot |
| 2365.1589 | 2365.1633 | 0.0044 | 2 | 598 | 624 | ASASGSGAQVGGPISSG SSASSVTVTR |  |  |  | Mascot |

**(P11142) Heat shock cognate 71 kDa protein HS7C_HUMAN 438 100 368 100 71082.3 5.37 22 1791962.12**

| \| **Peptide Information** \| \|  \| \| \| \| \| \| \| \| \| \| \| \| --- \| --- \| --- \| --- \| --- \| --- \| --- \| --- \| --- \| --- \| --- \| --- \| --- \| \| **Calc. Mass** \| **Obsrv. Mass** \| \| **± da** \| **± ppm** \| **Start**  **Seq.** \| **End**  **Seq.** \| **Sequence** \| **Ion**  **Score** \| **C. I. %** \| **Modification** \| **Rank** \| **Result Type** \| | | | | | | | | | | | | |  |  |
| --- | --- | --- | --- | --- | --- | --- | --- | --- | --- | --- | --- | --- | --- | --- | --- | --- | --- | --- | --- | --- | --- | --- | --- | --- | --- | --- | --- | --- | --- | --- | --- | --- | --- | --- | --- | --- | --- | --- | --- | --- |
|  | | | | | |  | | |  | | | |  |  |
|  | | | | | |  | | |  | | | |  |  |
|  | | | | | |  | | |  | | | |  |  |
|  | | | | | |  | | |  | | | |  |  |
|  | | | | | |  | | |  | | | |  |  |
|  | | | | | |  | | |  | | | |  |  |
|  | | | | | |  | | |  | | | |  |  |
| 905.4621 | 905.4636 | 0.0015 | 2 | 263 | | 269 | LRTACER | |  |  | Carbamidomethyl (C)[5] | | | Mascot |
| 985.4284 | 985.4409 | 0.0125 | 13 | 263 | | 269 | LRTACER | |  |  | Carbamidomethyl (C)[5], Phospho (ST)[3] | | | Mascot |
| 1141.5558 | 1141.5417 | -0.0141 | -12 | 518 | | 526 | MVQEAEKYK | |  |  | Oxidation (M)[1] | | | Mascot |
| 1199.6742 | 1199.6654 | -0.0088 | -7 | 160 | | 171 | DAGTIAGLNVLR | |  |  |  | | | Mascot |
| 1199.6742 | 1199.6654 | -0.0088 | -7 | 160 | | 171 | DAGTIAGLNVLR | | 81 | 100 |  | | | Mascot |
| 1253.6161 | 1253.6075 | -0.0086 | -7 | 302 | | 311 | FEELNADLFR | |  |  |  | | | Mascot |
| 1319.5936 | 1319.5819 | -0.0117 | -9 | 540 | | 550 | NSLESYAFNMK | |  |  | Oxidation (M)[10] | | | Mascot |
| 1407.7201 | 1407.7072 | -0.0129 | -9 | 237 | | 247 | MVNHFIAEFKR | |  |  | Oxidation (M)[1] | | | Mascot |
| 1480.7543 | 1480.7418 | -0.0125 | -8 | 300 | | 311 | ARFEELNADLFR | |  |  |  | | | Mascot |
| 1480.7543 | 1480.7418 | -0.0125 | -8 | 300 | | 311 | ARFEELNADLFR | | 58 | 99.998 |  | | | Mascot |
| 1487.7013 | 1487.6895 | -0.0118 | -8 | 37 | | 49 | TTPSYVAFTDTER | |  |  |  | | | Mascot |
| 1565.8322 | 1565.8082 | -0.024 | -15 | 349 | | 361 | LLQDFFNGKELNK | |  |  |  | | | Mascot |
| 1627.9126 | 1627.8872 | -0.0254 | -16 | 156 | | 171 | QATKDAGTIAGLNVLR | |  |  |  | | | Mascot |
| 1632.7826 | 1632.7705 | -0.0121 | -7 | 113 | | 126 | SFYPEEVSSMVLTK | |  |  | Oxidation (M)[10] | | | Mascot |
| 1691.7256 | 1691.7134 | -0.0122 | -7 | 221 | | 236 | STAGDTHLGGEDFDNR | |  |  |  | | | Mascot |
| 1787.9901 | 1787.979 | -0.0111 | -6 | 172 | | 188 | IINEPTAAAIAYGLDKK | | 76 | 100 |  | | | Mascot |
| 1787.9901 | 1787.979 | -0.0111 | -6 | 172 | | 188 | IINEPTAAAIAYGLDKK | |  |  |  | | | Mascot |
| 1821.8912 | 1821.8811 | -0.0101 | -6 | 57 | | 72 | NQVAMNPTNTVFDAKR | |  |  | Oxidation (M)[5] | | | Mascot |
| 1952.0599 | 1952.0436 | -0.0163 | -8 | 452 | | 469 | DNNLLGKFELTGIPPAPR | |  |  |  | | | Mascot |
| 1981.9978 | 1981.991 | -0.0068 | -3 | 138 | | 155 | TVTNAVVTVPAYFNDSQ R | | 152 | 100 |  | | | Mascot |
| 1981.9978 | 1981.991 | -0.0068 | -3 | 138 | | 155 | TVTNAVVTVPAYFNDSQ R | |  |  |  | | | Mascot |
| 1997.9457 | 1997.9559 | 0.0102 | 5 | 326 | | 342 | LDKSQIHDIVLVGGSTR | |  |  | Phospho (ST)[4,15] | | | Mascot |
| 2093.0549 | 2093.0469 | -0.008 | -4 | 302 | | 319 | FEELNADLFRGTLDPVEK | |  |  |  | | | Mascot |
| 2156.0869 | 2156.082 | -0.0049 | -2 | 37 | | 56 | TTPSYVAFTDTERLIGDA AK | |  |  |  | | | Mascot |
| 2774.3267 | 2774.353 | 0.0263 | 9 | 424 | | 447 | QTQTFTTYSDNQPGVLIQ  VYEGER | |  |  |  | | | Mascot |

**(P49411) Elongation factor Tu, mitochondrial precursor (EF-Tu) (P43) EFTU_HUMAN 78 99.71 59 99.999 49852.3 7.26 10 586777.25**

**(EF-TU)(P43)**

## Peptide Information

**pe**

| **Calc. Mass Obsrv. Mass** | | **± da** | **± ppm** | **Start**  **Seq.** | **End Sequence**  **Seq.** | | **Ion**  **Score** | **C. I. % Modification Rank Result Ty** | |
| --- | --- | --- | --- | --- | --- | --- | --- | --- | --- |
| 851.5097 | 851.5084 | -0.0013 | -2 | 163 | 169 | EHLLLAR |  |  | Mascot |
| 978.5115 | 978.5042 | -0.0073 | -7 | 422 | 429 | FTLRDGNR |  |  | Mascot |
| 1032.5684 | 1032.5736 | 0.0052 | 5 | 272 | 281 | GTVVTGTLER |  |  | Mascot |
| 1185.6222 | 1185.6111 | -0.0111 | -9 | 316 | 327 | AEAGDNLGALVR |  |  | Mascot |
| 1492.6914 | 1492.6755 | -0.0159 | -11 | 91 | 102 | KYEEIDNAPEER |  |  | Mascot |
| 1542.8527 | 1542.8329 | -0.0198 | -13 | 239 | 252 | LLDAVDTYIPVPAR |  |  | Mascot |
| 1670.882 | 1670.8617 | -0.0203 | -12 | 312 | 327 | SLERAEAGDNLGALVR |  |  | Mascot |
| 1673.8605 | 1673.8464 | -0.0141 | -8 | 105 | 120 | GITINAAHVEYSTAAR | 59 | 99.999 | Mascot |
| 1673.8605 | 1673.8464 | -0.0141 | -8 | 105 | 120 | GITINAAHVEYSTAAR |  |  | Mascot |
| 1677.8806 | 1677.8646 | -0.016 | -10 | 352 | 366 | VEAQVYILSKEEGGR |  |  | Mascot |
| 2129.1641 | 2129.1553 | -0.0088 | -4 | 253 | 271 | DLEKPFLLPVEAVYSVPG  R |  |  | Mascot |
